# Audiovestibular Dysfunction in Patients with Hashimoto’s Disease: A Systematic Review

**DOI:** 10.3390/ijms26104703

**Published:** 2025-05-14

**Authors:** Jiann-Jy Chen, Chih-Wei Hsu, Tien-Yu Chen, Chih-Sung Liang, Yen-Wen Chen, Bing-Yan Zeng, Ping-Tao Tseng

**Affiliations:** 1 Prospect Clinic for Otorhinolaryngology & Neurology, Kaohsiung 811, Taiwan; jiannjy@yahoo.com.tw (J.-J.C.); kevinachen0527@gmail.com (Y.-W.C.); 2 Department of Otorhinolaryngology, E-Da Cancer Hospital, I-Shou University, Kaohsiung 824, Taiwan; 3 Department of Psychiatry, Kaohsiung Chang Gung Memorial Hospital and Chang Gung University College of Medicine, Kaohsiung 833, Taiwan; harwicacademia@gmail.com; 4 Department of Psychiatry, Tri-Service General Hospital, School of Medicine, National Defense Medical Center, Taipei 114, Taiwan; verducciwol@gmail.com; 5 Institute of Brain Science, National Yang Ming Chiao Tung University, Taipei 112, Taiwan; 6 Department of Psychiatry, Beitou Branch, Tri-Service General Hospital, School of Medicine, National Defense Medical Center, Taipei 112, Taiwan; lcsyfw@gmail.com; 7 Department of Psychiatry, National Defense Medical Center, Taipei 114, Taiwan; 8 Institute of Biomedical Sciences, National Sun Yat-sen University, Kaohsiung 804, Taiwan; 9 Department of Internal Medicine, E-Da Dachang Hospital, I-Shou University, Kaohsiung 807, Taiwan; 10 Institute of Precision Medicine, National Sun Yat-sen University, Kaohsiung 804, Taiwan

**Keywords:** Hashimoto’s disease, cochleopathy, vestibular dysfunction, sensorineural hearing loss, treatment

## Abstract

Although the inner ear is considered an immune-privileged organ because of the blood–labyrinth barrier, accumulating evidence has revealed an unexpected relation between Hashimoto’s disease and inner ear damage manifesting as audiovestibular dysfunction. Hashimoto’s disease can simultaneously affect both the auditory and vestibular systems, either through direct autoantibody attacks or through metabolic dysfunction associated with hypothyroidism. Currently, there is no consensus regarding tests or treatments for audiovestibular dysfunction related to Hashimoto’s disease. In this review, we summarize the currently available evidence regarding the characteristics, pathophysiology, diagnostic approaches, and treatment of audiovestibular dysfunction in patients with Hashimoto’s disease. Furthermore, we propose a specific steroid-plus-thyroxine treatment protocol to manage audiovestibular dysfunction associated with Hashimoto’s disease. This condition may respond to adequate treatment, potentially allowing reversibility if it is recognized and managed in a timely manner. Conversely, delayed diagnosis or failure to recognize the subtle presentation of audiovestibular dysfunction in patients with Hashimoto’s disease may lead to progressive hearing loss, immobility, and reduced quality of life. Based on the updated evidence in our review and our modified treatment protocol, we aim to provide new insights and therapeutic directions for clinicians managing audiovestibular dysfunction in patients with Hashimoto’s disease. Trial registration: PROSPERO CRD420250652982.

## 1. Introduction

Hashimoto’s disease, in which autoantibodies target the thyroid gland, includes both Hashimoto’s thyroiditis and Hashimoto’s encephalopathy [1]. The key features of Hashimoto’s disease are the presence of antithyroid peroxidase (anti-TPO) and/or antithyroglobulin (anti-TG) antibodies, with the subsequent chronic lymphocytic infiltration or inflammation of the thyroid gland [1]. Hashimoto’s thyroiditis is one of the most common inflammatory thyroid disorders and is sometimes complicated by hypothyroidism [1].

Although the inner ear was previously considered an immune-privileged organ because of the blood–labyrinth barrier [2], recent evidence has demonstrated an unexpected relation between inner ear disorders and audiovestibular dysfunctions associated with various autoimmune diseases [3,4,5]. Among autoimmune inner ear diseases—which account for less than 1% of audiovestibular dysfunction cases [6]—autoimmune thyroid diseases, particularly Hashimoto’s disease, are recognized as major contributors. Audiovestibular dysfunction in patients with Hashimoto’s disease accounts for 17.9% of all autoimmune-mediated inner ear disorders [7]. Most research has emphasized the impact of thyroid hormone levels—a secondary consequence rather than a primary etiology of Hashimoto’s disease—on audiovestibular function. For example, Gu et al. [8], through a meta-analysis, found that patients with hypothyroidism had a higher risk of unspecified hearing loss than that of controls. However, only 20%–30% of patients with Hashimoto’s thyroiditis develop hypothyroidism [9]. Similarly, in a multicenter case–control study, most patients with autoimmune thyroiditis and benign paroxysmal positional vertigo were euthyroid (79%) rather than hypothyroid (21%) [10]. In another case–control study, hearing impairment was associated with the presence of antithyroid antibodies but not with abnormal thyroid function [11]. Furthermore, in a study primarily involving patients with Hashimoto’s thyroiditis, approximately 26.7% had concurrent cochlear and vestibular dysfunction [12].

Despite these findings, few studies have directly examined the relation between Hashimoto’s disease and audiovestibular dysfunction, regarding either auditory [7,11,13] or vestibular aspects [14,15]. Among these, a notable prevalence of vestibular dysfunction associated with autoimmune thyroiditis (27.1% of all patients with benign paroxysmal positional vertigo) was observed [15]. Similarly, approximately 46.4% of patients with Hashimoto’s thyroiditis were reported to experience hearing impairment [16]. Another report documented a broad deterioration of hearing function in patients with Hashimoto’s disease, initially affecting extended high frequencies (8–20 kHz) and progressing to all frequencies over time [11]. This finding is clinically important, as standard pure-tone audiometry in most countries only assesses frequencies between 128 and 8000 Hz [11], thereby potentially overlooking early-stage dysfunction. Consequently, the prevalence of audiovestibular dysfunction in patients with Hashimoto’s disease may be underestimated. Hashimoto’s disease not only complicates the course of auditory dysfunction but also worsens the prognosis of vestibular disorders. Specifically, Tricarico et al. reported that the presence of Hashimoto’s disease was significantly associated with recurrent benign paroxysmal positional vertigo [17]. Furthermore, elevated anti-TPO and anti-TG antibody titers were positively correlated with recurrence rates [17]. The audiovestibular symptoms associated with Hashimoto’s disease tend to follow a progressive course with fluctuating patterns, affecting both cochlear and vestibular functions [18].

Unlike other forms of idiopathic sensorineural hearing loss, audiovestibular dysfunction in patients with Hashimoto’s disease may respond to appropriate treatment [7,18], potentially enabling partial or full recovery if diagnosed and managed early. Furthermore, even when symptoms are refractory to therapy, treatment remains essential to slow disease progression. Therefore, to provide clinically relevant guidance to healthcare professionals, this systematic review summarizes the current evidence on the characteristics, pathophysiology, diagnostic assessment, and treatment of audiovestibular dysfunction in patients with Hashimoto’s disease.

## 2. Methods

### 2.1. Literature Search Strategy

This systematic review was conducted by electronically searching the PubMed, Embase, ClinicalKey, Web of Science, and ScienceDirect on-line platforms. The detailed search strategy and keywords used on each platform are listed in Table S2. Additionally, a manual literature search was performed to scrutinize the reference lists of the included articles. The final update search date was 16 February 2025. If there had been insufficient information available in the original paper, we contacted the corresponding authors via email to request data of interest.

### 2.2. Inclusion and Exclusion Criteria

The current systematic review aims to focus on audiovestibular issues, including their characteristics, pathophysiology, examination, and treatment, in patients with Hashimoto’s disease. Therefore, the inclusion criteria were as follows: (a) articles that examined the aforementioned audiovestibular issues in patients with Hashimoto’s disease; (b) articles that were case reports/series, observational trials, case–control trials, or randomized controlled trials; and (c) articles recruiting patients with Hashimoto’s disease.

The exclusion criteria were the following: (a) articles not recruiting patients with Hashimoto’s disease; (b) articles not related to the characteristics, pathophysiology, examination, or treatment related to audiovestibular dysfunction in patients with Hashimoto’s disease; and (c) animal studies. Review articles were chosen to manually extract articles from their reference list. The excluded articles are listed in Table S3.

### 2.3. Article Screening Process

After electronically searching all five databases with the inclusion/exclusion criteria, all of the search items would be screened by title and abstract. All eligible articles were downloaded and underwent full-text examination. In this stage, duplicate articles were removed manually, and the remaining articles were screened by full-text examination to determine whether they are to be included in this review.

### 2.4. Data Extraction

Data extraction was performed by P.T. Tseng and Y.W. Chen, who carried out full-text examinations and extracted data regarding the characteristics, pathophysiology, examination, and treatment of patients with Hashimoto’s disease.

### 2.5. Article Quality Grading

All clinical studies were graded by YW Chen and PT Tseng via the Newcastle–Ottawa Scale [19] (Table S4).

## 3. Results and Discussion

**Figure 1:**
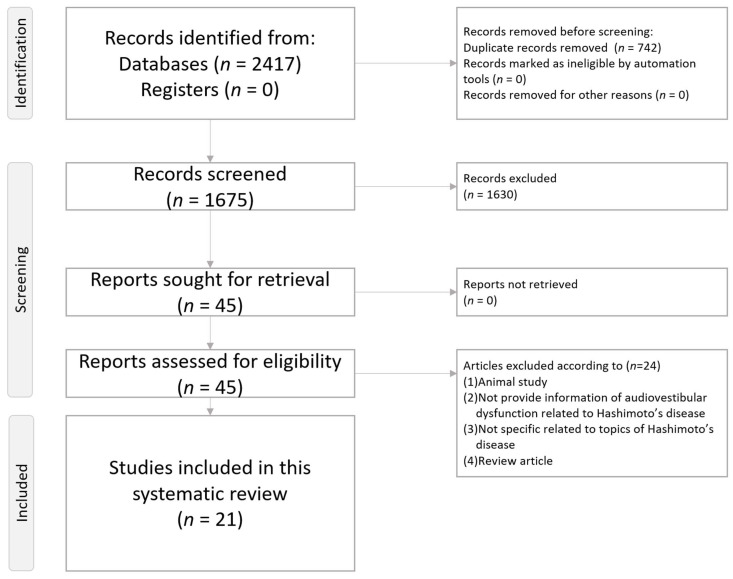
PRISMA2020 Flowchart of the current systematic review.

This systematic review follows the direction of the Preferred Reporting Items for Systematic Review and Meta-Analysis (PRISMA) statement (Table S1 and Figure 1) [20]. The current systematic review has been registered in PROSPERO with the number CRD420250652982.

Figure 1 depicts a flowchart illustrating the procedure of the present systematic review.

**Figure 2:**
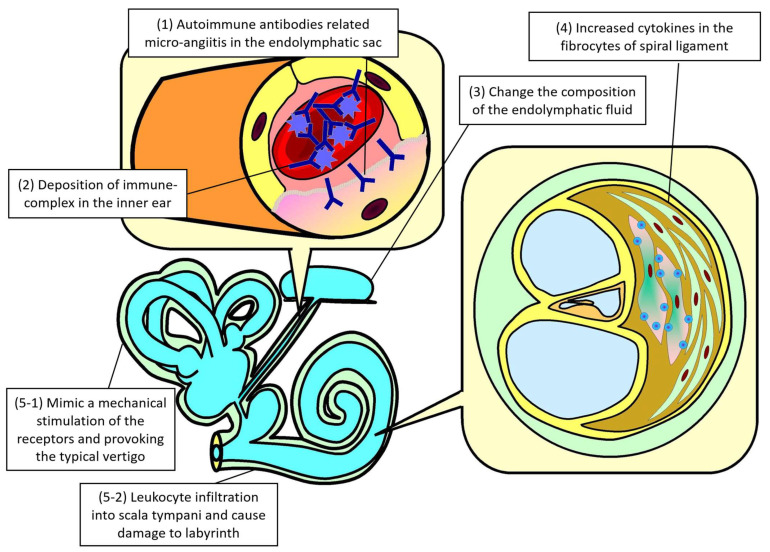
Schematic diagram of the physiopathology of Hashimoto’s disease in audiovestibular dysfunction.

This review article summarizes the current knowledge about audiovestibular dysfunction related to Hashimoto’s disease. A schematic of the overall pathophysiology of Hashimoto’s disease-associated audiovestibular impairment is shown in Figure 2. In brief, the pathophysiology of Hashimoto’s disease-associated audiovestibular dysfunction consists of several steps, including (1) microangiitis in the endolymphatic sac mediated by autoimmune antibodies, (2) immune complex deposition in the inner ear, (3) alteration of the endolymphatic fluid composition, (4) increased cytokine levels in the fibrocytes of the spiral ligament, (5-1) mechanical receptor stimulation mimicked by immunologic activity, provoking typical vertigo, and (5-2) leukocyte infiltration into the scala tympani, resulting in labyrinthine damage.

Figure 2 illustrates the pathophysiology of Hashimoto’s disease-associated audiovestibular dysfunction. It overall consisted of several steps, including (1) autoimmune antibody-related micro-angiitis in the endolymphatic sac, (2) deposition of the immune complex in the inner ear, (3) change in the composition of the endolymphatic fluid, (4) increased cytokines in the fibrocytes of spiral ligaments, (5-1) mimicking a mechanical stimulation of the receptors and provoking the typical vertigo, and (5-2) leukocyte infiltration into scala tympani, causing damage to the labyrinth.

Table 1 summarizes the characteristics of vestibular and auditory system involvement in patients with Hashimoto’s disease.

### 3.1. Vestibular System Involvement

#### 3.1.1. Characteristics of Vestibular System Involvement

Vestibular system involvement in Hashimoto’s disease is sometimes associated with Hashimoto’s encephalopathy, a variant of the disease [14]. This involvement frequently occurs in pediatric patients, whose vestibular systems are less mature than those of adult patients [14]. For example, Ueno et al. demonstrated that rotatory vertigo occurred in a full-term, normally developed boy [14]. This subacute vertigo persisted and progressed over an 11-month period and finally subsided after 4 months of immunoglobulin and steroid pulse therapy. Furthermore, vestibular symptoms are not necessarily associated with hypothyroidism. Papi et al. demonstrated the predominance of euthyroid states (79%) among patients with benign paroxysmal positional vertigo and autoimmune thyroiditis [10]. By contrast, 18% of patients with euthyroid Hashimoto’s thyroiditis presented with benign paroxysmal positional vertigo [21]. Benign paroxysmal positional vertigo primarily results from canalithiasis and cupulolithiasis, with the posterior semicircular canals most frequently affected [22].

#### 3.1.2. Pathophysiology of Vestibular System Involvement

Although no definitive conclusions have been reached regarding the pathophysiology of vestibular dysfunction related to Hashimoto’s disease, several plausible hypotheses have been proposed. Specifically, the deposition of immune complexes in the inner ear may alter the composition of the endolymphatic fluid, mimicking the mechanical stimulation of the receptors and inducing typical vertigo [15]. Additionally, autoimmune antibodies inducing microangiitis in the endolymphatic sac may support the classification of this condition within the spectrum of autoimmune-mediated multiorgan syndromes [21]. Consequently, antithyroid antibodies may reach the endolymphatic sac of the inner ear, impairing endolymphatic flow and leading to vertigo [23]. Clinical evidence supports this hypothesis. For instance, a largescale meta-analysis by Lima et al. demonstrated a statistically significant co-occurrence of Hashimoto’s thyroiditis—but not hypothyroidism—with benign paroxysmal positional vertigo [24]. Miskiewicz-Orczyk et al. found no association between thyroid hormone levels and cervical vestibular-evoked myogenic potentials or directional preponderance in caloric testing [25]. Collectively, vestibular dysfunction in patients with Hashimoto’s disease is most likely the result of an immune-mediated inflammatory cascade affecting receptor cells in the vestibular organ of the inner ear [25], rather than an effect of hypothyroidism itself [23].

Low levels of vitamin D are associated with elevated thyroid-stimulating hormone concentrations in end-stage Hashimoto’s disease [26]. Persistently low vitamin D levels increase the risk of osteoporosis, heighten susceptibility to positional vertigo, alter otolith structure, and promote the detachment of otoliths from the utricular macula [23].

Although rare, cerebral vasculitis associated with Hashimoto’s encephalopathy—confirmed through electroencephalography and single-photon emission-computed tomography—is another possible etiology of vestibular dysfunction related to Hashimoto’s disease [27]. For example, in a case report by Chen et al., symptoms, such as sudden-onset vertigo, diplopia, and recurrent gait ataxia, were linked to vasculitis associated with Hashimoto’s encephalopathy and responded well to steroid-plus-thyroxine therapy [27]. Given that thyroid gland dysfunction plays a minimal role in sensorineural hearing loss in autoimmune thyroid diseases [7], recent investigations into the pathophysiology of tinnitus in patients with Hashimoto’s cochleopathy have shifted toward the involvement of anti-TPO and anti-TG antibodies. These antibodies have been detected in cerebrospinal fluid [28,29], suggesting a potential link to Hashimoto’s encephalopathy. The condition may therefore not be directly related to thyroid dysfunction but rather to antibody-mediated attacks [30]. These autoimmune antibodies can bind to non-thyroid antigens in the brain, leading to vascular occlusion, T cell-mediated vestibulocochlear dysfunction, and cytotoxic effects on endothelial cells mediated by anti-TPO and anti-TG antibodies [18,31].

#### 3.1.3. Examination of Vestibular System Involvement

As vestibular involvement is sometimes associated with Hashimoto’s encephalopathy, the instruments used in the detection of Hashimoto’s encephalopathy could help detect vestibular dysfunction related to Hashimoto’s disease. For example, a positive anti-TPO antibody result combined with evidence of cortical dysfunction (i.e., epileptic spikes or slow waves on electroencephalogram and diffuse insufficient blood flow on brain single-photon emission computed tomography) could be an indicator of the presence of Hashimoto’s encephalopathy regardless of thyroid function [14].

Furthermore, across different thyroid-related antibodies, the anti-TPO antibody is one of the most important antibodies that shows a consistent association with audiovestibular diseases related to Hashimoto’s disease [24,32].

The assistance of videonystagmography, which has been widely used in the diagnosis of vestibular organ disease, could help define three-dimensional nystagmus in patients with Hashimoto’s disease [23]. Although nonspecific, other traditional vertigo tests, such as the oculomotor test, caloric response test, video head impulse test, and cervical vestibular-evoked myogenic potentials, could help distinguish central-origin vertigo [33].

### 3.2. Auditory System Involvement

#### 3.2.1. Characteristics of Auditory System Involvement

Auditory system involvement, characterized by unilateral or bilateral sensorineural hearing loss, is one of the most common otologic symptoms of Hashimoto’s disease [7]. Among patients with hearing loss, sudden-onset sensorineural hearing loss was the most frequent clinical presentation (53.3%) [7]. Tinnitus, one of the symptoms related to hearing loss, has been found to be associated with thyroid diseases (17.7% of patients with tinnitus also had thyroid disease) [34]. Conversely, participants with hypothyroidism had a higher risk of tinnitus than those without hypothyroidism [35]. Similar findings were reported in a previous case [18]. In the report by Álvarez Montero et al., the authors identified a statistically significant hearing impairment at all frequencies in patients with Hashimoto’s thyroiditis compared with that in controls, which was more profound at 9–16 kHz and 20 kHz in the 20–49 years age group [11]. Therefore, they concluded that sensorineural hearing loss related to Hashimoto’s disease initially affected extended high frequencies in pure-tone audiometry; however, it evolved to all frequencies as the disease progressed, such that it could be detected in routine clinical tests [11]. The affected frequency of hearing function varies at different stages across the disease course [36]. In another report by Arduc et al., the authors demonstrated a high prevalence of sensorineural hearing loss at 250, 500, and 6000 Hz, with a positive correlation with antithyroid antibody levels in a middle-aged group of patients with Hashimoto’s disease [36]. When focusing on the ordinary frequency ranges of pure-tone audiometry, this disease duration-dependent evolution was not observed. Specifically, in another report by Gunes et al., the authors found no significant difference between 250 and 8000 Hz frequencies in patients with 1-year disease duration versus those with 5-year disease duration [37].

#### 3.2.2. Physiopathology of Auditory System Involvement

Although the actual pathophysiology of Hashimoto’s disease-associated audiological dysfunction remains unclear, Hashimoto et al. presented an animal model of cochlear damage related to the adaptive innate immune response that mimics the etiology of immune-mediated inner ear disease in humans [38]. The activation of the innate immune response is considered one of the etiologies of autoimmune thyroiditis [39]. During the innate immune response, the upregulation of cytokines (i.e., interleukin-1β) in the fibrocytes of the spiral ligament and infiltration of leukocytes into the scala tympani also enhance the damage to the labyrinth [38].

In addition to the anti-TPO antibody theory, the potential involvement of the pendrin protein might also be one of the mechanisms underlying Hashimoto’s disease-associated audiovestibular dysfunction, as it is expressed in both the thyroid gland and inner ear, especially the stria vascularis [7]. Although rarely tested in clinical practice, anti-pendrin antibodies—such as anti-TG and anti-TPO antibodies—are correlated with autoantibodies related to Hashimoto’s disease [7]. Dysfunctional pendrin could serve as one of the first steps in immune-mediated inner ear diseases, with features of dysfunctional stria vascularis and spiral ligament [7]. Dysfunction in these structures affects the supporting cells of the organ of Corti, and consequently, hair cells, leading to the development of audiovestibular symptoms [40]. Although rare, comorbid Pendred syndrome—a genetic disease characterized by sensorineural hearing loss and thyroid goiter [41]—and suspected Hashimoto’s thyroiditis might sometimes co-occur [42].

If Hashimoto’s disease progresses to hypothyroidism, the hormone-dependent damage in the auditory system would worsen hearing loss. Specifically, the consequent late-stage hypothyroidism affects hearing function, owing to the impaired metabolism and oxygenation of the organ of Corti and stria vascularis [12], which leads to dysfunctional protein synthesis, cell growth and differentiation, myelin production, and electrolyte imbalance [11]. The impact of hypothyroidism mainly contributes to sensorineural but rarely to conductive or mixed hearing loss, depending on the site of damage [43]. The etiology of hypothyroidism-related conductive hearing impairment may result from middle ear mucosal edema caused by hypothyroidism [44].

#### 3.2.3. Examination of Auditory System Involvement

Since it is clinically irrelevant to retrieve histopathological evidence through biopsy of the inner ear, laboratory tests using peripheral blood autoimmune profiles and other noninvasive tests have become the major examination tools for detecting autoimmune labyrinthitis [45]. Although not specific or sensitive for Hashimoto’s disease in patients with auditory system involvement, several investigative tools can detect sensorineural hearing loss. In the clinical study by Topaloğlu et al., the authors observed that pure-tone audiometry revealed significantly (a) higher thresholds in both ears from 250 to 8000 Hz, (b) less negative tympanic peak pressure, (c) a higher proportion of negative acoustic reflex testing, and (d) more abnormal transient-evoked otoacoustic emissions in patients with euthyroid Hashimoto’s disease than in controls [13]. Abnormal findings on pure-tone audiometry can be symmetric or asymmetric, with either sensorineural or mixed hearing loss patterns [18]. Furthermore, tympanic peak pressure and air conduction thresholds are significantly and positively correlated with anti-TPO antibody titers, indicating a potential pathophysiological link between these parameters [13]. In a pediatric study of euthyroid Hashimoto’s disease, the authors reported considerable disturbances in the auditory nerve and brainstem neural conduction based on brain auditory-evoked potentials [46]. Similar abnormal findings in auditory-evoked potentials were also observed in another report of an adult patient with audiovestibular dysfunction related to Hashimoto’s encephalopathy [47].

Conversely, as addressed in the report by Álvarez Montero et al. [11], the sensorineural hearing loss related to Hashimoto’s disease initially predominates at extended high frequencies in pure-tone audiometry and progresses to affect all frequencies as the disease advances. As mentioned previously, the ordinary frequency ranges (i.e., 250 to 8000 Hz) of pure-tone audiometry are not effective in the early detection of hearing impairments related to Hashimoto’s disease [37]. Therefore, the early and routine application of extended high-frequency audiometry in the diagnosis of subclinical hearing loss in patients with Hashimoto’s thyroiditis is clinically relevant and important, especially in those aged 20–49 years [11]. Furthermore, although the authors did not exclusively recruit patients with Hashimoto’s disease, significantly increased wave V absolute latencies in brainstem auditory-evoked potential testing and decreased transient otoacoustic emission amplitudes were observed in a study in which 70% of the patients were diagnosed with Hashimoto’s thyroiditis [12].

Peripheral blood examination of abnormal immune profiles is a major diagnostic tool for Hashimoto’s disease-associated audiovestibular dysfunction. To fulfill the diagnosis of Hashimoto’s disease, a positive anti-TPO antibody result, with or without abnormal thyroid function, is the key finding. Dysfunctional T regulatory cells and T cell-related cell lines also play an important role in the development of immune-mediated inner ear disease [48,49,50].

### 3.3. Treatment of Hashimoto’s Disease-Related Audiovestibular Dysfunction

Currently, no specific treatment is available for audiovestibular dysfunction related to Hashimoto’s disease. Instead, treatment primarily focuses on managing the underlying Hashimoto’s disease, which typically involves modalities such as steroid therapy and high-dose intravenous immunoglobulin.

Steroid therapy, which is usually administered as pulse therapy, is considered an evidence-based treatment for all types of sensorineural hearing loss [51], including that associated with Hashimoto’s disease. Intratympanic steroid therapy is frequently used for Ménière’s disease, which is significantly associated with thyroid dysfunctions [52,53]. Additional systemic corticosteroid therapy based on high-dose intravenous immunoglobulin improved vestibular symptoms related to pediatric Hashimoto’s encephalopathy [14]. Similarly, in a case report by Fayyaz et al., after the timely recognition of Hashimoto’s disease-associated hearing loss, oral prednisone (60 mg once daily) improved symptoms after 1 week, with full resolution following the completion of a 3-week treatment course [18]. Regarding the timing of steroid discontinuation, Rodríguez-Valiente et al. suggested that tapering or withdrawing steroids in steroid-responsive cases might be less risky and less likely to result in relapse [7]. However, some patients still become steroid-dependent, although no definitive data exist on the prevalence of steroid dependency in such individuals [27,47].

Thyroxine supplementation, which is commonly used in patients with hypothyroidism, is theoretically considered a treatment option for Hashimoto’s disease-associated audiovestibular dysfunction. Considering the aforementioned relation between hypothyroidism and hearing impairment, hormone replacement therapy with thyroxine may improve hearing thresholds in patients with hypothyroidism, especially those with mild disease [54]. Evidence has also suggested the potential roles of triiodothyronine [55] and certain genetic conditions [56]. However, in the report by Rodríguez-Valiente et al., the authors reported an unsatisfactory response to thyroxine supplementation in audiovestibular dysfunction related to Hashimoto’s disease [7]. Steroid therapy—either oral or intratympanic—provides more favorable efficacy for hearing recovery, since autoimmune disease is the underlying mechanism of Hashimoto’s disease-associated audiovestibular dysfunction [7]. Nevertheless, in a report by Malik et al., hearing threshold improvement in 30% of ears was attributed to thyroxine supplementation in patients with hypothyroidism, based on the hypothesis of cochlear and tectorial membrane dysfunction caused by hypothyroid status [57]. However, Malik et al. did not specifically address Hashimoto’s disease, which has distinct characteristics in the majority of euthyroid patients.

Other treatment regimens, such as rituximab, antagonists of T helper cells, and chemokine blockers [58], have not been recommended for the management of audiovestibular dysfunction related to Hashimoto’s disease, although they have been applied in other immune-mediated inner ear diseases [7]. Since concerns about ototoxicity and uncertain efficacy have not been resolved, the risk–benefit profile of such medications remains inconclusive.

Finally, although less common, Giribet Fernandez-Pacheco et al., in a small-scale retrospective study, observed that patients with Hashimoto’s disease, either in euthyroid or hypothyroid states, achieved better vertigo control after receiving thyroidectomy [59]. However, because thyroidectomy is invasive and irreversible, it should only be performed in patients with refractory audiovestibular dysfunction related to Hashimoto’s disease when the disease is unresponsive to conservative therapy.

#### 3.3.1. Temporary Protocol of Steroid Treatment to Manage Hashimoto’s Disease-Associated Audiovestibular Dysfunction

**Figure 3:**
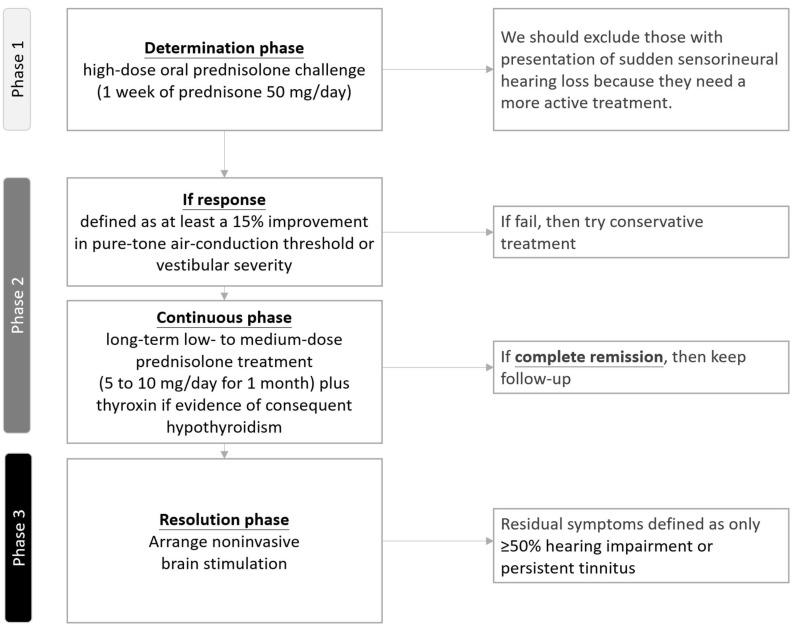
Flowchart of the modified steroid treatment protocol to manage Hashimoto’s disease-associated audiovestibular dysfunction.

In our preliminary study, we developed a modified steroid treatment protocol (Figure 3) to specifically manage Hashimoto’s disease-associated audiovestibular dysfunction, which is distinct from other autoimmune inner ear diseases. This protocol, based on our previous protocol for various autoimmune inner ear diseases, consists of a three-phase treatment [3,4,5].

Figure 3 shows the modified steroid treatment protocol that focuses on the management of audiovestibular dysfunction related to Hashimoto’s disease. Note: This is a proposal for future study protocols.

We conducted a three-phase trial. In phase 1, patients underwent a high-dose oral prednisolone trial (1 week of prednisolone 50 mg/d) to determine their response to steroid treatment. Response was defined as at least 15% improvement in the pure-tone air-conduction threshold or vestibular symptom severity (according to any vestibular symptom severity rating scale). Following phase 1, patients began long-term low- to medium-dose prednisolone treatment (5–10 mg/d for 1 month) in phase 2. Furthermore, in phase 2, thyroxine is added depending on the presence or absence of consequent hypothyroidism, which differs from other autoimmune inner ear diseases. Finally, in phase 3, patients with residual symptoms (≥50% hearing impairment or persistent tinnitus) undergo audiovestibular dysfunction-specific noninvasive brain stimulation [60,61].

#### 3.3.2. Recommendations About Referral to Otorhinolaryngological Examination

**Figure 4:**
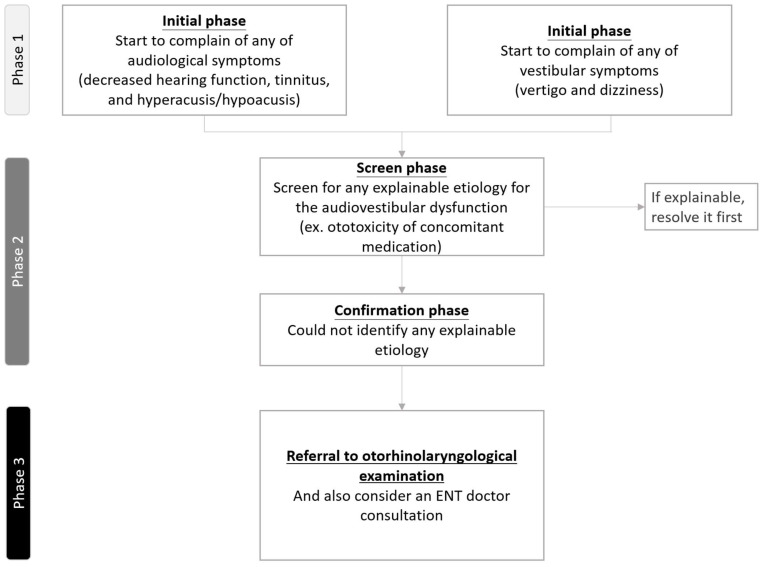
Flowchart of recommendations about referral to otorhinolaryngological examination.

Although there is no consensus regarding the red flags or timing of referral for otorhinolaryngological examination, we have summarized some potential time points for referral consideration based on our previous report and clinical practice experience [4]. First, patients complain of audiological symptoms, such as decreased hearing function, tinnitus, or hyperacusis/hypoacusis. Second, patients report unexplained vestibular symptoms, such as vertigo and dizziness. Third, clinicians should consider ENT referral if a patient’s audiovestibular symptoms are unlikely to be related to the ototoxicity of a prescribed medication. Finally, although subjective, clinicians should consider ENT referral if they notice that their clinical advice is frequently ignored by a specific patient. A detailed flowchart of the referral process is shown in Figure 4.

Figure 4 demonstrates the recommended referral flowchart for any complaints of audiovestibular symptoms.

## 4. Conclusions

Through the available evidence, one undeniable fact emerges as follows: unrecognized Hashimoto’s disease-associated audiovestibular dysfunction can ultimately lead to generalized hearing loss, patient immobility, and impaired quality of life. As mentioned earlier, the subtle presentation of audiovestibular dysfunction related to Hashimoto’s disease underscores the importance of clinicians’ awareness of the potential risk of vestibular and auditory system involvement in asymptomatic patients with Hashimoto’s disease. Certain audiometric and vestibular tests can aid in the early detection of audiovestibular impairment in patients with Hashimoto’s disease. Therefore, the extensive utilization of these tools and routine checkups for hearing and vestibular function should be conducted in patients with Hashimoto’s disease to prevent further audiovestibular deterioration.

Moreover, unlike other idiopathic autoimmune-mediated inner ear diseases, audiovestibular dysfunction related to Hashimoto’s disease may respond to appropriate treatments and may be reversible if promptly recognized and managed. Therefore, we strongly advocate for the early and routine screening of audiovestibular function in such patients, even during asymptomatic stages.

### Limitation

There were several limitations to this study, including the lack of direct pathological evidence to confirm Hashimoto’s disease-associated audiovestibular dysfunction, limited number of articles included, and limited evidence regarding the proposed treatment protocol.

## Figures and Tables

**Table 1:** Summary of the characteristics of vestibular system involvement and auditory system involvement.

Characteristics	Vestibular System Involvement	Auditory System Involvement
Clinical Feature	Sometimes associated with Hashimoto’s encephalopathy, especially in pediatric subjects.	Unilateral or bilateral sensorineural hearing loss. Predominantly sudden onset sensorineural hearing loss.
Hypothyroidism	Not necessarily associated with the presence of hypothyroidism.	Sometimes associated with hypothyroidism in a reciprocal relationship.
Clinical Examination	Mainly resulted from canalithiasis and cupulolithiasis, among which the posterior semicircular canals were the most often affected parts. Recording of cortical dysfunction evidence plus the existence of positive anti-thyroid peroxidase antibody. Traditional videonystagmography, oculomotor test, caloric response test, video head impulse test, and cervical vestibular-evoked myogenic potentials, could help in distinguishing central-origin or peripheral-origin vertigo	Hearing impairment in all frequencies. More profound between 9–16 kHz and 20 kHz in young age patients. Sensorineural hearing loss may have a positive correlation with anti-thyroid antibody levels. Ordinary frequency ranges of pure-tone audiometry are not recommended. Abnormal findings in brain auditory-evoked potential. Tympanic peak pressure and air conduction thresholds were significantly positively correlated with the anti-TPO antibody titers

## Supplementary Materials

The following supporting information can be downloaded at: https://www.mdpi.com/article/10.3390/ijms26104703/s1. References [62,63,64,65,66,67,68,69,70,71,72] are cited in the Supplementary Materials.
