# Supplementary material for: Audiovestibular Dysfunction in Patients with Hashimoto’s Disease: A Systematic Review
Source: Int J Mol Sci. 2025 May 14;26(10):4703. doi: 10.3390/ijms26104703 (PMC12111619; doi:10.3390/ijms26104703)
Supplement: Supplementary file 1 [file ijms-26-04703-s001.zip › ijms-3567463-supplementary.pdf]

Table S1: PRISMA 2020 checklist of current systematic review

Section and Topic	Item #	Checklist item	Page where item is reported
**TITLE**			
Title	1	Identify the report as a systematic review.	1
**ABSTRACT**			
Abstract	2	See the PRISMA 2020 for Abstracts checklist.	4
**INTRODUCTION**			
Rationale	3	Describe the rationale for the review in the context of existing knowledge.	6-7
Objectives	4	Provide an explicit statement of the objective(s) or question(s) the review addresses.	6-7
**METHODS**			
Eligibility criteria	5	Specify the inclusion and exclusion criteria for the review and how studies were grouped for the syntheses.	8-9
Information sources	6	Specify all databases, registers, websites, organisations, reference lists and other sources searched or consulted to identify studies. Specify the date when each source was last searched or consulted.	8-9
Search strategy	7	Present the full search strategies for all databases, registers and websites, including any filters and limits used.	8-9
Selection process	8	Specify the methods used to decide whether a study met the inclusion criteria of the review, including how many reviewers screened each record and each report retrieved, whether they worked independently, and if applicable, details of automation tools used in the process.	8-9
Data collection process	9	Specify the methods used to collect data from reports, including how many reviewers collected data from each report, whether they worked independently, any processes for obtaining or confirming data from study investigators, and if applicable, details of automation tools used in the process.	8-9
Data items	10a	List and define all outcomes for which data were sought. Specify whether all results that were compatible with each outcome domain in each study were sought (e.g. for all measures, time points, analyses), and if not, the methods used to decide which results to collect.	8-9
	10b	List and define all other variables for which data were sought (e.g. participant and intervention characteristics, funding sources). Describe any assumptions made about any missing or unclear information.	9-10
Study risk of bias assessment	11	Specify the methods used to assess risk of bias in the included studies, including details of the tool(s) used, how many reviewers assessed each study and whether they worked independently, and if applicable, details of automation tools used in the process.	9-10
Effect measures	12	Specify for each outcome the effect measure(s) (e.g. risk ratio, mean difference) used in the synthesis or presentation of results.	9-10
Synthesis methods	13a	Describe the processes used to decide which studies were eligible for each synthesis (e.g. tabulating the study intervention characteristics and comparing against the planned groups for each synthesis (item #5)).	Not done
	13b	Describe any methods required to prepare the data for presentation or synthesis, such as handling of missing summary statistics, or data conversions.	Not done

Section and Topic	Item #	Checklist item	Page where item is reported
	13c	Describe any methods used to tabulate or visually display results of individual studies and syntheses.	Not done
	13d	Describe any methods used to synthesize results and provide a rationale for the choice(s). If meta-analysis was performed, describe the model(s), method(s) to identify the presence and extent of statistical heterogeneity, and software package(s) used.	Not done
	13e	Describe any methods used to explore possible causes of heterogeneity among study results (e.g. subgroup analysis, meta-regression).	Not done
	13f	Describe any sensitivity analyses conducted to assess robustness of the synthesized results.	Not done
Reporting bias assessment	14	Describe any methods used to assess risk of bias due to missing results in a synthesis (arising from reporting biases).	9-10
Certainty assessment	15	Describe any methods used to assess certainty (or confidence) in the body of evidence for an outcome.	9-10
**RESULTS**			
Study selection	16a	Describe the results of the search and selection process, from the number of records identified in the search to the number of studies included in the review, ideally using a flow diagram.	11-12
	16b	Cite studies that might appear to meet the inclusion criteria, but which were excluded, and explain why they were excluded.	11-12
Study characteristics	17	Cite each included study and present its characteristics.	11-12
Risk of bias in studies	18	Present assessments of risk of bias for each included study.	11-12
Results of individual studies	19	For all outcomes, present, for each study: (a) summary statistics for each group (where appropriate) and (b) an effect estimate and its precision (e.g. confidence/credible interval), ideally using structured tables or plots.	11-12
Results of syntheses	20a	For each synthesis, briefly summarise the characteristics and risk of bias among contributing studies.	Not done
	20b	Present results of all statistical syntheses conducted. If meta-analysis was done, present for each the summary estimate and its precision (e.g. confidence/credible interval) and measures of statistical heterogeneity. If comparing groups, describe the direction of the effect.	Not done
	20c	Present results of all investigations of possible causes of heterogeneity among study results.	Not done
	20d	Present results of all sensitivity analyses conducted to assess the robustness of the synthesized results.	Not done
Reporting biases	21	Present assessments of risk of bias due to missing results (arising from reporting biases) for each synthesis assessed.	13-14
Certainty of evidence	22	Present assessments of certainty (or confidence) in the body of evidence for each outcome assessed.	13-14
**DISCUSSION**			
Discussion	23a	Provide a general interpretation of the results in the context of other evidence.	16-18
	23b	Discuss any limitations of the evidence included in the review.	16-18
	23c	Discuss any limitations of the review processes used.	16-18

Section and Topic	Item #	Checklist item	Page where item is reported
	23d	Discuss implications of the results for practice, policy, and future research.	19
**OTHER INFORMATION**			
Registration and protocol	24a	Provide registration information for the review, including register name and registration number, or state that the review was not registered.	5
	24b	Indicate where the review protocol can be accessed, or state that a protocol was not prepared.	5
	24c	Describe and explain any amendments to information provided at registration or in the protocol.	5
Support	25	Describe sources of financial or non-financial support for the review, and the role of the funders or sponsors in the review.	20
Competing interests	26	Declare any competing interests of review authors.	20
Availability of data, code and other materials	27	Report which of the following are publicly available and where they can be found: template data collection forms; data extracted from included studies; data used for all analyses; analytic code; any other materials used in the review.	20

The current checklist followed the latest PRISMA 2020 guideline.[1]

Table S2: Keyword and search results in each database

Database	Keyword	Filter	Date	Result
PubMed	(Hashimoto thyroiditis) AND (hearing loss OR sensorineural hearing loss OR SNHL OR audiology OR tinnitus OR vertigo OR vestibular OR dizziness)	N/A	2025/02/15	121
Embase	(Hashimoto thyroiditis) AND (hearing loss OR sensorineural hearing loss OR SNHL OR audiology OR tinnitus OR vertigo OR vestibular OR dizziness)	N/A	2025/02/15	421
ClinicalKey	(Hashimoto thyroiditis) AND (hearing loss OR sensorineural hearing loss OR SNHL OR audiology OR tinnitus OR vertigo OR vestibular OR dizziness)	N/A	2025/02/15	398
Web of Science	(Hashimoto thyroiditis) AND (hearing loss OR sensorineural hearing loss OR SNHL OR audiology OR tinnitus OR vertigo OR vestibular OR dizziness)	N/A	2025/02/15	37
ScienceDirect	(Hashimoto thyroiditis) AND (hearing loss OR sensorineural hearing loss OR SNHL OR audiology OR tinnitus OR vertigo OR vestibular OR dizziness)	N/A	2025/02/15	1440

Abbreviation: N/A: not applied

Table S3: Excluded studies and reason

Reason	Numbers	References
Animal study	1	[2]
Not provide information of audiovestibular dysfunction related to Hashimoto's disease	8	[3-10]
Not specific related to topics of Hashimoto's disease	13	[11-23]
Review article	2	[24,25]

Table S4: Newcastle-Ottawa Scale and Characteristics for the Included Trial

Study	Selection				Comparability	Exposure			Total
	Case definition	Representative	Control selection	Control definition	Comparability	Ascertainment	Same method	Non-Response rate	Summary
Miura, A. (2024)[26]	*					*			2*
Seymen, G. (2024)[27]	*	*	*	*	*	*	*		7*
Álvarez Montero, O.L. (2023)[28]	*	*	*	*	*	*	*		7*
Lima, C.M. (2023)[29]	*		*		*	*			4*
Miskiewicz-Orczyk, K. (2022)[30,31]	*	*				*			3*
Topaloğlu, O. (2022)[32]	*	*	*	*	*	*	*		7*
Giribet Fernández-Pacheco A (2021)[33]	*	*				*			3*
Rodríguez-Valiente, A. (2019)[34]	*	*	*	*	*	*	*		7*
Fayyaz, B. (2018)[35]	*					*			2*
Kong, F.X. (2018)[36]	*					*			2*
Gunes, A. (2017)[37]	*	*	*	*	*	*	*		7*

Ueno, H. (2016)[38]	*					*		2*
Arduc, A. (2015)[39]	*	*	*	*	*	*	*	7*
Renda, L. (2015)[40]	*	*	*	*	*	*	*	7*
Chiarella, G. (2014)[41]	*	*	*	*	*	*	*	7*
Papi, G. (2010)[42]	*	*	*	*	*	*	*	7*
Papi, G. (2009)[43]	*	*	*	*	*	*	*	7*
Chen, P.L. (2005)[44]	*					*		2*
Gawron, W. (2004)[45]	*	*				*		3*
Modugno, G.C. (2000)[46]	*	*	*	*	*	*	*	7*

* indicated this study have a good performance in this item

Table S5: Summary of the included study

Study	Characteristics<br>Study design	Recruited case characteristics	Outcomes<br>Results	Summary<br>Conclusion
Miura, A. (2024)[26]	Case report	A girl with both suspected Pendred syndrome and suspected Hashimoto’s thyroiditis.	Genetic test revealed *SLC26A4* variants. Laboratory data revealed positive anti-thyroid peroxidase antibody.	Evaluating the comorbidity of Hashimoto's thyroiditis with the SLC26A4 variant in terms of complications is critical.
Seymen, G. (2024)[27]	Case-control	Thirty-six children with Hashimoto’s thyroiditis and 30 healthy subjects were recruited. The main inclusion criteria for the healthy group were the presence of normal thyroid function and no clinical history of vestibular problems.	There was no significant difference between the groups in terms of cervical-evoked myogenic potentials latencies or amplitudes between the groups. There were statistically significant differences in the vestibulo-ocular reflex gain of the right and left ears between the groups.	Subclinical vestibulopathy may occur in children with Hashimoto’s thyroiditis. The video head impulse test seems to be a valuable tool for identifying and screening preclinical vestibular pathologies in this patient group.
Álvarez Montero, O.L. (2023)[28]	Case-control	Hashimoto’s thyroiditis subjects with/without thyroxin supplement	Statistically significant hearing impairment in all frequencies in Hashimoto’s thyroiditis subjects compared to controls, which was more profound in 9−16 kHz and 20 kHz in young group (20-49 y/o).	Hearing loss related to the autoimmune disorder predominates at extended-high-frequencies initially but ends up involving all frequencies.
Lima, C.M. (2023)[29]	Meta-analysis	Subjects with thyroid disease, either Hashimoto’s disease or other cause, and benign paroxysmal positional vertigo.	The meta-analysis showed a statistically significant relationship between Hashimoto thyroiditis, but not hypothyroidism, and benign paroxysmal positional vertigo.	The meta-analysis results suggest a possible association between benign paroxysmal positional vertigo and Hashimoto thyroiditis.
Miskiewicz-Orczyk, K. (2022)[30,31]	Single arm case study	Twenty eight women with Hashimoto's thyroiditis and chronic vertigo were enrolled	No correlation was found between age, weight, height, BMI and the results of thyroid function tests or the objective assessment of the vestibular organ	The study did not confirm the influence of thyroid metabolism (i.e., thyroid hormone levels or the increase in antithyroid antibodies) on the results of cVEMP or the directional preponderance in the caloric test
Topaloğlu, O. (2022)[32]	Case-control	Adult patients with euthyroid Hashimoto’s disease (normal thyroid functions, positive antithyroid	(a) Higher both ears threshold from 250 to 8,000 Hz, (b) less negative tympanic peak pressure, (c) a higher ratio of negative acoustic reflex testing, and (d) more abnormal transient evoked	Hearing and audiological tests may be impaired in euthyroid Hashimoto’s disease. We recommend close

		peroxidase/anti-thyroglobulin, antibody, and sonographic findings)	otoacoustic emission in subjects with euthyroid Hashimoto’s disease than controls	monitoring of audiological functions in these patients
Giribet Fernández-Pacheco A (2021)[33]	Single-arm case study	52 patients who presented Hashimoto’s thyroiditis were recruited.	Patients with vestibular symptoms had laboratory evidence of hypothyroidism. Most of them had increased levels of anti-peroxidase and anti-thyroglobulin antibodies.	Results show an increased prevalence of hypothyroidism in patients with vestibular dysfunction and better control of vertigo after thyroidectomy.
Rodríguez-Valiente, A. (2019)[34]	Case-control	Focusing on adult Hashimoto’s disease patients (i.e. positive antithyroid peroxidase with/without abnormal thyroid function)	Thyroxin supplementation provided an unsatisfactory response on audiovestibular dysfunction related to Hashimoto’s disease. Rather, steroid therapy, either oral or intra-tympanic injection, provided a preferable efficacy of hearing recovery.	In the present study, although all the patients with hypothyroidism and subclinical hypothyroidism were being treated with levothyroxine, immune-mediated hearing loss was observed. Therapy with corticosteroids could achieve hearing recovery
Fayyaz, B. (2018)[35]	Case report	A 61-year-old female with a history of Hashimoto’s thyroiditis with positive antithyroid peroxidase antibodies complained of progressive hearing loss	3-week prednisone 60 mg once daily completely resolve the hearing loss symptoms	It should be considered in the differential diagnosis of hearing disorders especially in the context of another autoimmune disease such as Hashimoto's thyroiditis.
Kong, F.X. (2018)[36]	Case report	A case of a 63-year-old female patient with paroxysmal dizziness, unsteady gait, emotion apathy, progressive cognitive impairment related to Hashimoto's encephalopathy.	Continued regular glucocorticoids therapy after intravenous methylprednisolone pulse therapy relieved clinical symptoms.	Hashimoto's encephalopathy may cause highly heterogeneous clinical features. Withdrawal of the systematic glucocorticoids treatment can lead to varied outcomes in these patients.
Gunes, A. (2017)[37]	Case-control	Study population consisted of 17 patients who had thyroid antibody positivities for an average of 1 year (first group), 15 patients who were followed up for at least 5 years for thyroid antibody positivity (second group), and 18 volunteers (third group).	Significant impaired hearing frequencies between 500 and 4000 Hz was found in case groups in comparison with controls. However, no significant difference between two case groups.	Since these effects become deeper in line with increased exposure to autoantibody positivity, variations in auditory responses should be more attentively monitored.
Ueno, H. (2016)[38]	Case report	A boy with progressively declined vestibular function accompanied with	Positive antithyroid peroxidase antibody plus evidence of cortical dysfunction (i.e. epileptic spike or slow waves on electroencephalogram and	Systemic corticosteroid therapy and high-dose intravenous immunoglobulin

		evidence of Hashimoto's encephalopathy	diffused insufficient blood flow in brain single photon emission computed tomography)	therapy were effective, greatly improving his quality of life
Arduc, A. (2015)[39]	Case-control	30 patients with newly diagnosed euthyroid Hashimoto's thyroiditis and 30 age- and gender-matched healthy controls.	Higher audiometric thresholds and a higher prevalence of hearing loss at 250, 500, and 6000 Hz were detected in the Hashimoto's thyroiditis patients. Hearing levels at 250 and 500 Hz correlated positively with anti-thyroglobulin antibody levels	This study demonstrated that hearing functions are impaired in Hashimoto's thyroiditis patients. Thyroid autoimmunity seems to have an important impact on a decreased hearing ability, particularly at lower frequencies, in this population of patients.
Renda, L. (2015)[40]	Case-control	Children and adolescents aged 9-18 years with diagnosed of Hashimoto's thyroiditis	Distortion product otoacoustic emissions signal to noise ratios (6-1.5K Hz) were significantly lower in the case group. More signal to noise ratios at all frequencies in case group. Distortion product amplitudes were significantly lower in the case group.	Cochlear function was lower in the case group than in the control group. Hearing in patients with Hashimoto's thyroiditis should be monitored periodically, even if their hearing thresholds are within normal limits.
Chiarella, G. (2014)[41]	Case-control	Forty-seven patients with Hashimoto's thyroiditis and 30 healthy volunteers were enrolled.	52.2% of Hashimoto's thyroiditis patients showed an alteration of vestibular evoked myogenic potentials and 44.7% of caloric test, while only one healthy control showed an altered caloric test. A correlation was found between vestibular alterations and the degree of serum antithyroid peroxidase autoantibody level	In euthyroid Hashimoto's thyroiditis patients, a significant relationship between subclinical vestibular damage and the degree of antithyroid peroxidase autoantibody titre was documented.
Papi, G. (2010)[42]	Case-control	Subjects with euthyroid Hashimoto's thyroiditis and matched healthy controls	In subjects with euthyroid Hashimoto's thyroiditis, 18% had symptoms of benign paroxysmal positional vertigo.	Authors suggested that thyroid autoimmunity be always checked in the work-up of patients with benign paroxysmal positional vertigo.
Papi, G. (2009)[43]	Case-control	Cases with history of benign paroxysmal positional vertigo and matched healthy controls.	Significantly higher anti-thyroid antibodies in subjects with benign paroxysmal positional vertigo. Among them, 21% were hypothyroid and 79% euthyroid.	Benign paroxysmal positional vertigo is strongly associated with both hypothyroidism and thyroid autoimmunity.

Chen, P.L. (2005)[44]	Case report	Case 3 was an elderly male with Hashimoto's encephalopathy who presented with a sudden onset of vertigo, diplopia, and recurrent gait ataxia.	Symptoms subsided under oral prednisolone 30 mg/day control. However, symptoms fluctuated after discontinuation of steroid.	Vasculitis is the most probable pathogenesis according to laboratory findings. Steroids are the treatment of choice, and result in a favorable outcome when administered early in the disease course.
Gawron, W. (2004)[45]	Single arm case study	Thirty children with euthyroid Hashimoto's thyroiditis	Pure tone audiometry, tympanometry, and otoacoustic emissions were normal in all patients. There were considerable disturbances in auditory nerve and brainstem neural conduction in brain auditory evoked potentials.	One should consider the possible presence of subclinical Hashimoto's encephalopathy affecting the central part of the auditory
Modugno, G.C. (2000)[46]	Case-control	Cases with history of benign paroxysmal positional vertigo. Among them, some had positive anti-thyroid antibodies.	About 27.1% of benign paroxysmal positional vertigo subjects had high anti-thyroid antibodies titers	The deposition of immune-complexes in inner ear changed the composition of the endolymphatic fluid.

Reference list of supplement tables:

1. Page, M.J.; McKenzie, J.E.; Bossuyt, P.M.; Boutron, I.; Hoffmann, T.C.; Mulrow, C.D.; Shamseer, L.; Tetzlaff, J.M.; Akl, E.A.; Brennan, S.E.; et al. The PRISMA 2020 statement: an updated guideline for reporting systematic reviews. *Bmj* **2021**, *372*, n71, doi:10.1136/bmj.n71.
2. Hashimoto, S.; Billings, P.; Harris, J.P.; Firestein, G.S.; Keithley, E.M. Innate immunity contributes to cochlear adaptive immune responses. *Audiol Neurootol* **2005**, *10*, 35-43, doi:10.1159/000082306.
3. Ragusa, F.; Fallahi, P.; Elia, G.; Gonnella, D.; Paparo, S.R.; Giusti, C.; Churilov, L.P.; Ferrari, S.M.; Antonelli, A. Hashimotos' thyroiditis: Epidemiology, pathogenesis, clinic and therapy. *Best Pract Res Clin Endocrinol Metab* **2019**, *33*, 101367, doi:10.1016/j.beem.2019.101367.
4. Kaur, J.; Jialal, I. Hashimoto Thyroiditis. In *StatPearls*; Treasure Island (FL) ineligible companies. Disclosure: Ishwarlal Jialal declares no relevant financial relationships with ineligible companies., 2025.
5. Hahn, H.J.; Kwak, S.G.; Kim, D.K.; Kim, J.Y. A Nationwide, Population-based Cohort Study on Potential Autoimmune Association of Meniere Disease to Atopy and Vitiligo. *Scientific reports* **2019**, *9*, 4406, doi:10.1038/s41598-019-40658-8.
6. Kang, M.Y.; Hahm, J.R.; Jung, T.S.; Lee, G.W.; Kim, D.R.; Park, M.H. A 20-year-old woman with Hashimoto's thyroiditis and Evans' syndrome. *Yonsei medical journal* **2006**, *47*, 432-436, doi:10.3349/ymj.2006.47.3.432.
7. Resende de Paiva, C.; Gronhoj, C.; Feldt-Rasmussen, U.; von Buchwald, C. Association between Hashimoto's Thyroiditis and Thyroid Cancer in 64,628 Patients. *Front Oncol* **2017**, *7*, 53, doi:10.3389/fonc.2017.00053.
8. Vanderpump, M.P. The epidemiology of thyroid disease. *Br Med Bull* **2011**, *99*, 39-51, doi:10.1093/bmb/ldr030.
9. Akay, B.N.; Bozkir, M.; Anadolu, Y.; Gullu, S. Epidemiology of vitiligo, associated autoimmune diseases and audiological abnormalities: Ankara study of 80 patients in Turkey. *J Eur Acad Dermatol Venereol* **2010**, *24*, 1144-1150, doi:10.1111/j.1468-3083.2010.03605.x.
10. Hsieh, C.T.; Yu, J.T.; Tsao, T.Y.; Tseng, Y.H. Mucosa-associated lymphoid tissue lymphoma of thyroid and adrenal glands with primary adrenal insufficiency. *Endocrinol Diabetes Metab Case Rep* **2024**, *2024*, doi:10.1530/EDM-23-0019.
11. Gu, L.; Guo, W.; Wang, X.; Yue, C. The association between thyroid disease and hearing loss: a meta-analysis. *Acta Otolaryngol* **2024**,

144, 495-502, doi:10.1080/00016489.2024.2404614.
12. Malik, V.; Shukla, G.K.; Bhatia, N. Hearing profile in hypothyroidism. *Indian J Otolaryngol Head Neck Surg* **2002**, *54*, 285-290, doi:10.1007/BF02993744.
13. Garcia-Berrocal, J.R.; Ramirez-Camacho, R.; Trinidad, A.; Zurita, M.; de la Fuente, R.; Lobo, D. Controversies and criticisms on designs for experimental autoimmune labyrinthitis. *Ann Otol Rhinol Laryngol* **2004**, *113*, 404-410, doi:10.1177/000348940411300512.
14. Berrocal, J.R.; Ramirez-Camacho, R. Sudden sensorineural hearing loss: supporting the immunologic theory. *Ann Otol Rhinol Laryngol* **2002**, *111*, 989-997, doi:10.1177/000348940211101107.
15. Bossowski, A.; Moniuszko, M.; Dabrowska, M.; Sawicka, B.; Rusak, M.; Jeznach, M.; Wojtowicz, J.; Bodzenta-Lukaszyk, A.; Bossowska, A. Lower proportions of CD4+CD25(high) and CD4+FoxP3, but not CD4+CD25+CD127(low) FoxP3+ T cell levels in children with autoimmune thyroid diseases. *Autoimmunity* **2013**, *46*, 222-230, doi:10.3109/08916934.2012.751981.
16. Garcia-Berrocal, J.R.; Vargas, J.A.; Ramirez-Camacho, R.A.; Gonzalez, F.M.; Gea-Banacloche, J.C.; Vergara, J.; Durantez, A. Deficiency of naive T cells in patients with sudden deafness. *Arch Otolaryngol Head Neck Surg* **1997**, *123*, 712-717, doi:10.1001/archotol.1997.01900070056009.
17. Ramirez-Camacho, R.; Garcia-Berrocal, J.R.; Trinidad, A.; Gonzalez-Garcia, J.A.; Verdaguer, J.M.; Ibanez, A.; Rodriguez, A.; Sanz, R. Central role of supporting cells in cochlear homeostasis and pathology. *Med Hypotheses* **2006**, *67*, 550-555, doi:10.1016/j.mehy.2006.02.044.
18. Rehman, U.L.; Hamza, H.M.; Malik, M.M.; Awan, A.A. Association of latent autoimmune diabetes of adults with type 3 polyglandular autoimmune syndrome-a diagnostic challenge. *J Pak Med Assoc* **2024**, *74*, 990-992, doi:10.47391/JPMA.10108.
19. Santos, K.T.; Dias, N.H.; Mazeto, G.M.; Carvalho, L.R.; Lapate, R.L.; Martins, R.H. Audiologic evaluation in patients with acquired hypothyroidism. *Braz J Otorhinolaryngol* **2010**, *76*, 478-484, doi:10.1590/S1808-86942010000400012.
20. Singh, R.; Aftab, M.; Jain, S.; Kumar, D. Audiological Evaluation in Hypothyroid Patients and Effect of Thyroxine Replacement Therapy. *Indian J Otolaryngol Head Neck Surg* **2019**, *71*, 548-552, doi:10.1007/s12070-018-1395-3.
21. Tricarico, L.; Di Cesare, T.; Galli, J.; Fetoni, A.R.; Paludetti, G.; Picciotti, P.M. Benign paroxysmal positional vertigo: is hypothyroidism a risk factor for recurrence? *Acta Otorhinolaryngol Ital* **2022**, *42*, 465-470, doi:10.14639/0392-100X-N1775.

22. Jain, M.; Shastri, M.; Patel, N.S.; Dobariya, R. Unravelling a Triad: A Rare Case of Takayasu Arteritis, Hashimoto's Thyroiditis, and Psoriasis in a Young Female With Heart Failure. *Cureus* **2024**, *16*, e61153, doi:10.7759/cureus.61153.
23. Choi, H.G.; Song, Y.S.; Wee, J.H.; Min, C.; Yoo, D.M.; Kim, S.Y. Analyses of the Relation between BPPV and Thyroid Diseases: A Nested Case-Control Study. *Diagnostics (Basel)* **2021**, *11*, doi:10.3390/diagnostics11020329.
24. Miskiewicz-Orczyk, K.A.; Lisowska, G.; Kajdaniuk, D.; Wojtulek, M. Can Hashimoto's thyroiditis cause vertigo? [Czy choroba Hashimoto moze byc przyczyna zawrotow glowy?]. *Endokrynol Pol* **2020**, *70*, 76-86, doi:10.5603/EP.a2019.0069.
25. Chiarella, G.; Russo, D.; Monzani, F.; Petrolo, C.; Fattori, B.; Pasqualetti, G.; Cassandro, E.; Costante, G. Hashimoto Thyroiditis and Vestibular Dysfunction. *Endocr Pract* **2017**, *23*, 863-868, doi:10.4158/EP161635.RA.
26. Miura, A.; Nakagawa, T.; Sogi, C.; Shima, H.; Adachi, M.; Honkura, Y.; Kikuchi, A.; Kanno, J. Hearing loss with two pathogenic SLC26A4 variants and positive thyroid autoantibody: A case report. *Clin Pediatr Endocrinol* **2024**, *33*, 219-223, doi:10.1297/cpe.2023-0084.
27. Seymen, G.; Gunay, G.; Cirik, A.A.; Surmeli, R.; Surmeli, M. Vestibular Dysfunction in Euthyroid Children with Hashimoto's Thyroiditis. *J Int Adv Otol* **2024**, *20*, 426-430, doi:10.5152/iao.2024.231123.
28. Alvarez Montero, O.L.; Rodriguez Valiente, A.; Gorriz Gil, C.; Garcia Berrocal, J.R. Audiological evaluation (128-20,000Hz) in women with autoimmune thyroiditis: The role of antibodies vs. l-thyroxine deficiency. *Acta Otorrinolaringol Esp (Engl Ed)* **2023**, *74*, 50-58, doi:10.1016/j.otoeng.2021.11.005.
29. Lima, C.M.; Paiva, D.F.F.; Corona, A.P.; Lessa, M.M. Association between Benign Paroxysmal Positional Vertigo and Thyroid Diseases: Systematic Review and Meta-Analysis. *Int Arch Otorhinolaryngol* **2024**, *28*, e530-e536, doi:10.1055/s-0043-1769496.
30. Miskiewicz-Orczyk, K.; Vlaykov, A.; Lisowska, G.; Strzelczyk, J.; Kos-Kudla, B. Does Thyroid Hormone Metabolism Correlate with the Objective Assessment of the Vestibular Organ in Patients with Vertigo? *J Clin Med* **2022**, *11*, doi:10.3390/jcm11226771.
31. Miskiewicz-Orczyk, K.; Kos-Kudla, B.; Lisowska, G. The function of the vestibular organ in Hashimoto's thyroiditis. *Endokrynol Pol* **2022**, *73*, 935-941, doi:10.5603/EP.a2022.0076.
32. Topaloglu, O.; Sahin, B. Hearing Impairment and Audiological Alterations in Euthyroid Hashimoto's Thyroiditis. *ORL J Otorhinolaryngol Relat Spec* **2022**, *84*, 238-246, doi:10.1159/000517931.

33. Giribet Fernandez-Pacheco, A.; Tomas Perez, M.A.; Almela Rojo, M.T.; Garcia-Purrinos Garcia, F.J. Relationship between vestibular syndrome and autoimmune thyroiditis. *Endocrinol Diabetes Nutr (Engl Ed)* **2021**, doi:10.1016/j.endinu.2020.09.007.
34. Rodriguez-Valiente, A.; Alvarez-Montero, O.; Gorriz-Gil, C.; Garcia-Berrocal, J.R. l-Thyroxine does not prevent immunemediated sensorineural hearing loss in autoimmune thyroid diseases. *Acta Otorrinolaringol Esp (Engl Ed)* **2019**, *70*, 229-234, doi:10.1016/j.otorri.2018.06.001.
35. Fayyaz, B.; Upreti, S. Autoimmune inner ear disease secondary to Hashimoto's thyroiditis: a case report. *J Community Hosp Intern Med Perspect* **2018**, *8*, 227-229, doi:10.1080/20009666.2018.1503917.
36. Kong, F.X.; Lu, Q.H.; Guo, Z.K. Multiple intracranial lesions as the unusual imaging features of Hashimoto's encephalopathy: A case report. *Medicine* **2018**, *97*, e10814, doi:10.1097/MD.0000000000010814.
37. Gunes, A.; Karakus, M.F.; Telli, T.A.; Gunes, N.A.; Mutlu, M. The effect of thyroid autoantibody positivity on the functions of internal ear. *Eur Arch Otorhinolaryngol* **2017**, *274*, 3853-3858, doi:10.1007/s00405-017-4723-4.
38. Ueno, H.; Nishizato, C.; Shimazu, T.; Watanabe, H.; Mizukami, T.; Kosuge, H.; Ozasa, S.; Nomura, K.; Kimura, S.; Takahashi, Y. [Hashimoto's encephalopathy presenting with vertigo and muscle weakness in a male pediatric patient]. *No To Hattatsu* **2016**, *48*, 45-47.
39. Arduc, A.; Isik, S.; Allusoglu, S.; Iriz, A.; Dogan, B.A.; Gocer, C.; Tuna, M.M.; Berker, D.; Guler, S. Evaluation of hearing functions in patients with euthyroid Hashimoto's thyroiditis. *Endocrine* **2015**, *50*, 708-714, doi:10.1007/s12020-015-0624-8.
40. Renda, L.; Parlak, M.; Selcuk, O.T.; Renda, R.; Eyigor, H.; Yilmaz, M.D.; Osma, U.; Filiz, S. Do antithyroid antibodies affect hearing outcomes in patients with pediatric euthyroid Hashimoto's thyroiditis? *Int J Pediatr Otorhinolaryngol* **2015**, *79*, 2043-2049, doi:10.1016/j.ijporl.2015.09.006.
41. Chiarella, G.; Tognini, S.; Nacci, A.; Sieli, R.; Costante, G.; Petrolo, C.; Mancini, V.; Guzzi, P.H.; Pasqualetti, G.; Cassandro, E.; et al. Vestibular disorders in euthyroid patients with Hashimoto's thyroiditis: role of thyroid autoimmunity. *Clin Endocrinol (Oxf)* **2014**, *81*, 600-605, doi:10.1111/cen.12471.
42. Papi, G.; Guidetti, G.; Corsello, S.M.; Di Donato, C.; Pontecorvi, A. The association between benign paroxysmal positional vertigo and autoimmune chronic thyroiditis is not related to thyroid status. *Thyroid* **2010**, *20*, 237-238, doi:10.1089/thy.2009.0319.

43. Papi, G.; Corsello, S.M.; Milite, M.T.; Zanni, M.; Ciardullo, A.V.; Donato, C.D.; Pontecorvi, A. Association between benign paroxysmal positional vertigo and autoimmune chronic thyroiditis. *Clin Endocrinol (Oxf)* **2009**, *70*, 169-170, doi:10.1111/j.1365-2265.2008.03311.x.
44. Chen, P.L.; Wang, P.Y.; Hsu, H.Y. Reversible electroencephalographic and single photon emission computed tomography abnormalities in Hashimoto's encephalopathy. *J Chin Med Assoc* **2005**, *68*, 77-81, doi:10.1016/S1726-4901(09)70139-X.
45. Gawron, W.; Pospiech, L.; Noczynska, A.; Orendorz-Fraczkowska, K. Electrophysiological tests of the hearing organ in Hashimoto's disease. *J Pediatr Endocrinol Metab* **2004**, *17*, 27-32, doi:10.1515/jpem.2004.17.1.27.
46. Modugno, G.C.; Pirodda, A.; Ferri, G.G.; Montana, T.; Rasciti, L.; Ceroni, A.R. A relationship between autoimmune thyroiditis and benign paroxysmal positional vertigo? *Med Hypotheses* **2000**, *54*, 614-615, doi:10.1054/mehy.1999.0905.
